# Supplementary material for: Characterization of Three Variants of SARS-CoV-2 In Vivo Shows Host-Dependent Pathogenicity in Hamsters, While Not in K18-hACE2 Mice
Source: Viruses. 2022 Nov 21;14(11):2584. doi: 10.3390/v14112584 (PMC9693146; doi:10.3390/v14112584)
Supplement: Supplementary file 1 [file viruses-14-02584-s001.zip › viruses-2025066-supplementary.pdf]

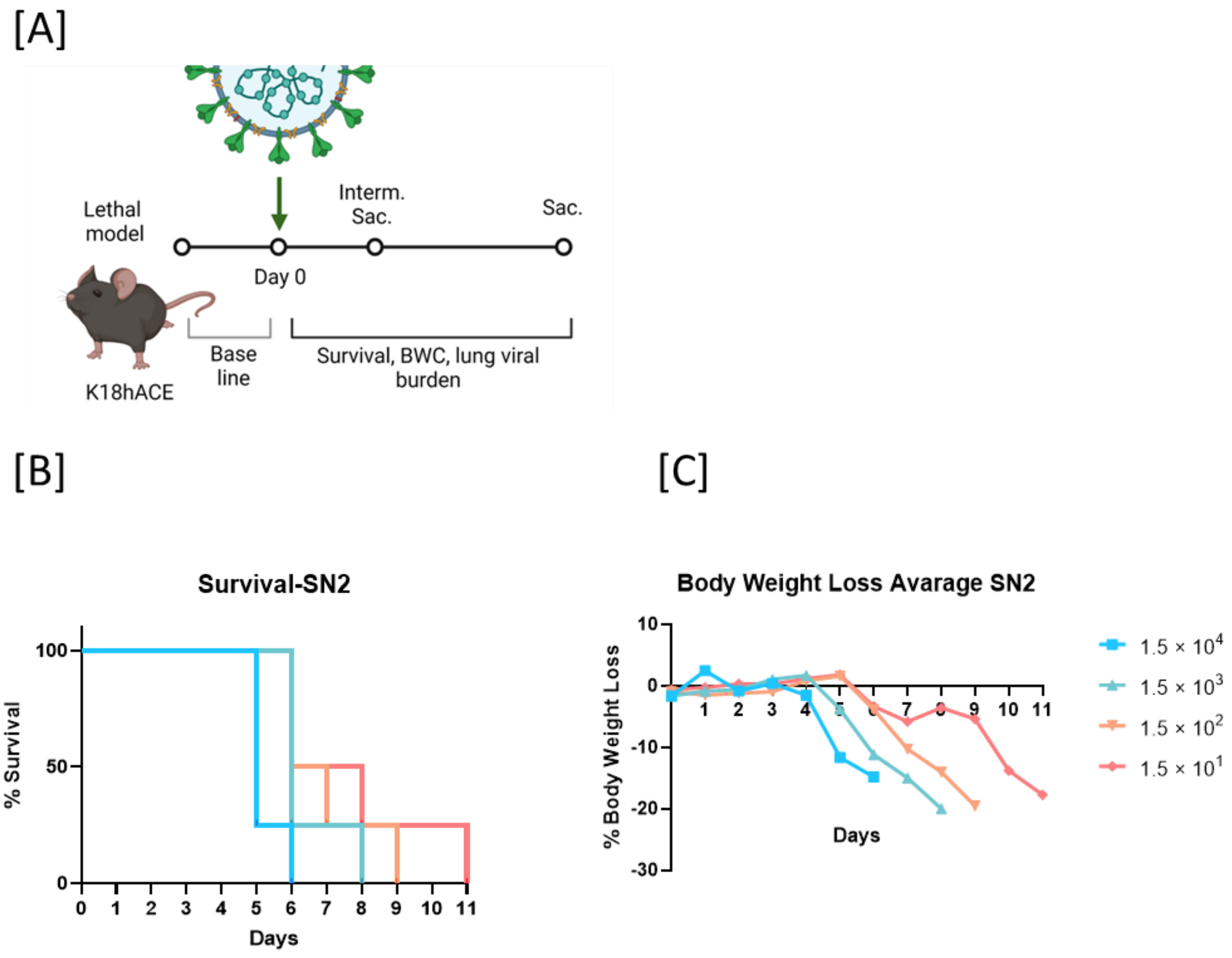

Supplemental Figure S1. Survival in K18-human angiotensin-converting enzyme 2 (hACE2) transgenic mice. (A) Experimental Design in mice (n = 6) infected intranasally with four (4) 10-fold serial dilutions of B.1.617.2/Delta SARS-CoV-2. Survival (B) and Body weight Loss (C) were monitored daily for 11 dpi**.**

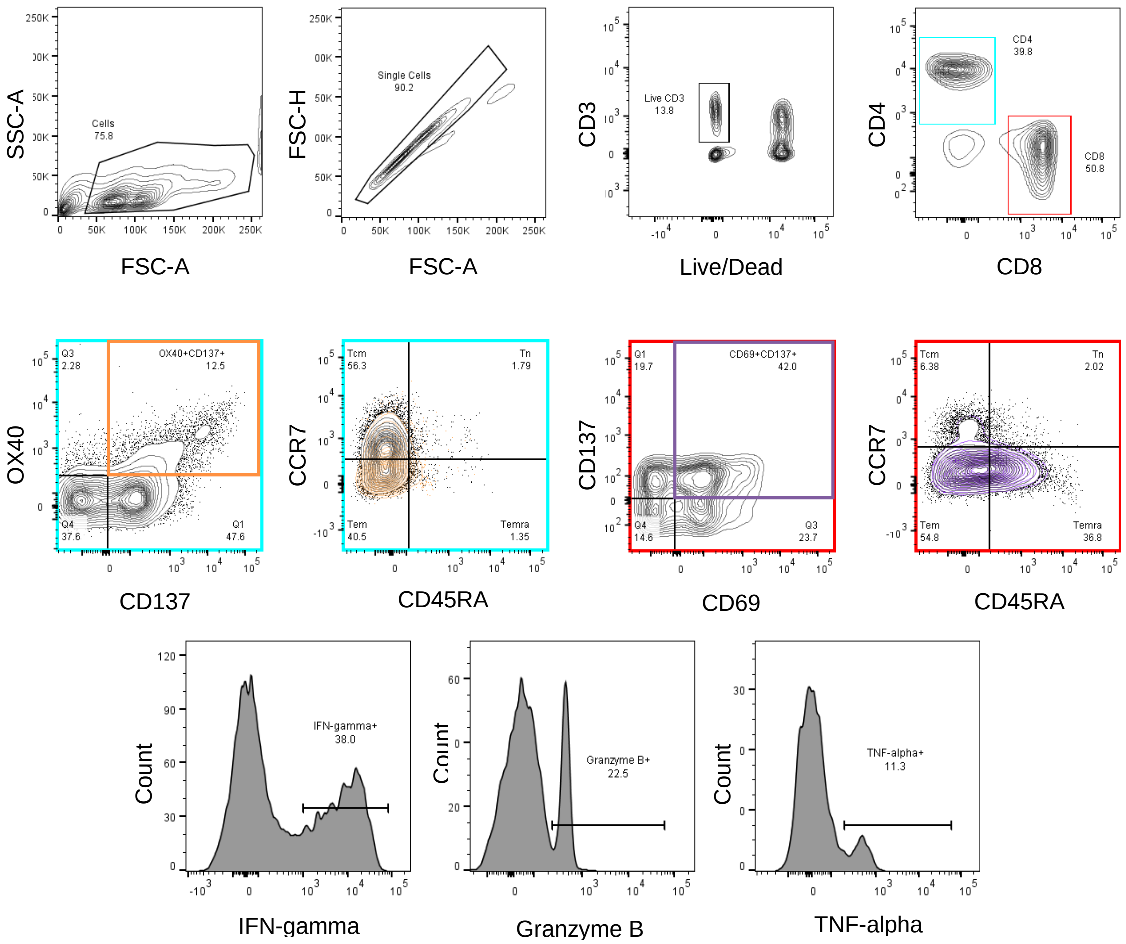

Supplemental Figure S2. Flow Cytometry Gating Strategy for AIM assay, memory phenotyping, and cytokine production. Representative graphs illustrating gating strategy used in flow cytometry-based assays to define antigen-specific CD4+ (outlined in blue) and CD8+ (outlined in red) T cells by the expression of OX40 +CD137+ and CD69+CD137+, respectively. The memory compartment was analyzed by gating naïve (Tn; CD45RA +CCR7+), central memory (Tcm; CD45RA-CCR7+), effector memory (Tem; CD45RA-CCR7-), and terminally differentiated effector memory (Temra; CD45RA+CCR7-) cells on bulk CD4+ and CD8+ T cells as well as AIM+ subsets, overlaid in orange and purple, respectively. Cytokine production was analyzed from both CD4+AIM+ and CD8+AIM+ T cells.
